# An immune challenge induces a decline in parental effort and compensation by the mate

**DOI:** 10.1093/beheco/arae086

**Published:** 2024-10-10

**Authors:** Alejandro Martínez-Flores, Bibiana Montoya, Roxana Torres

**Affiliations:** Laboratorio de Conducta Animal, Departamento de Ecología Evolutiva, Instituto de Ecología Universidad Nacional Autónoma de México, Circuito exterior Jardín Botánico, Ciudad de México, CP 04510, México; Programa de Posgrado en Ciencias Biológicas, Universidad Nacional Autónoma de México, 04510 Ciudad de México, México; Estación Científica La Malinche, Centro Tlaxcala de Biología de la Conducta (CTBC), Universidad Autónoma de Tlaxcala, Tlaxcala, CP 90070, México; Laboratorio de Conducta Animal, Departamento de Ecología Evolutiva, Instituto de Ecología Universidad Nacional Autónoma de México, Circuito exterior Jardín Botánico, Ciudad de México, CP 04510, México

**Keywords:** brown booby, immune challenge, LPS, parental effort, parental compensation, trade-off

## Abstract

Immune defense is fundamental to diminish the negative effects of the attack of infectious agents, yet the activation of the immune system entails costs and may compromise other life-history traits such as reproduction. In reproductive brown booby pairs (*Sula leucogaster*), we experimentally imposed an immune challenge during incubation, by intraperitoneally injecting *Escherichia coli* lipopolysaccharide (LPS), in either the male or the female. We aimed to test whether activation of the immune response results in (1) an increase in oxidative stress parameters, (2) a decline in post-hatching parental care in the treated individual, and (3) a compensation of the post-hatching parental effort by the nontreated mate. We found that activation of the immune response during incubation did not increase oxidative damage to lipids or total antioxidant capacity. However, mounting an immune response compromised parental effort during the chick-rearing period: compared to controls, LPS-treated parents showed roughly a 50% decline in the rate of preening and offspring feeding in response to begging. Interestingly, mates of LPS-treated parents increased their feeding rate suggesting parental care compensation. According to a scenario of full compensation, the decline in parental effort of LPS-treated parents did not result in poorer offspring growth or immune response, or increased levels of oxidative stress parameters. These findings suggest that in a long-lived species with long-lasting biparental care, an immune challenge compromises parental care, favoring parental compensation as a strategy to mitigate costs in terms of offspring success.

## Teaser

Defense against infection may influence the behavior of the host and other family members. In the brown booby, when one member of a breeding pair was immune challenged, we did not find increased levels of oxidative stress, a potential physiological cost of the immune response. However, parents who felt ill reduced their parental contribution, and their mates compensated for this reduction, avoiding negative effects on offspring.

## Introduction

Immune defenses are critical for survival, as individuals are frequently exposed to a wide diversity of parasites and pathogens, but this protection often comes at a cost (Sheldon and Verhulst 1996; Lochmiller and Deerenberg 2000). The activation of the immune response to resist infections can impose physiological and energetic costs to the host that may negatively impact other life-history components such as reproduction (Sheldon and Verhulst 1996; Moret and Schmid-Hempel 2000; Zuk and Stoehr 2002; Uller et al. 2006; Kulaszewicz et al. 2017). Accordingly, there is evidence of a trade-off between immune response and reproduction, as mounting an immune response during a reproductive attempt has been found to compromise parental care (Williams et al. 1999; Råberg et al. 2000; Bonneaud et al. 2003; Grzȩdzicka 2018), fledgling number and quality (Ilmonen et al. 2000), and long-term survival of the challenged parent (Hanssen et al. 2004; Graham et al. 2010). However, under ecologically relevant conditions, the physiological mechanisms underpinning these trade-offs are still uncertain. Furthermore, the potential consequences of mounting an immune response on the distribution of parental contribution to offspring care have been rarely investigated (Granroth-Wilding et al. 2015). Here, we evaluated in the brown booby, if oxidative stress may mediate the immediate physiological costs of mounting an immune response and the potential impact of immune activation on parental contribution to care.

Oxidative stress has been suggested as a key factor mediating the costs of immune activation (Costantini 2008; Costantini and Møller 2009; Hasselquist and Nilsson 2012). Oxidative stress is defined as an imbalance between reactive oxygen species and antioxidants in favor of the former, this imbalance increases the risk of suffering oxidative damage to biomolecules (Halliwell and Gutteridge 2007; Finkel 2011). Reactive oxygen species also play a cellular signaling role and are fundamental for the action of immune cells (phagocytes and lymphocytes) against invading pathogens (Sorci and Faivre 2009; Finkel 2011; Costantini 2014; Morris et al. 2022). However, the participation of reactive oxygen species in the immune response is not pathogen-specific and they could also damage the host cells as a side effect (Costantini 2014; Li et al. 2021). Hence, baseline antioxidant protection has been proposed to play a central role in modulating inflammatory response to an immune challenge, allowing faster recovery following an immune activation (Sorci and Faivre 2009; Cram et al. 2015).

An additional indirect cost of investing in immunity may arise if mounting an immune response changes the amount of resources that individuals contribute to parental care, impacting the social dynamics among family members (Knowles et al. 2010; Cotter et al. 2013; Granroth- Wilding et al. 2015). In socially monogamous species with biparental care, the cost of mounting an immune response may influence parental provisioning decisions of the immune-challenged individual and, as a consequence, the parental contribution of the nonchallenged partner, with potential fitness effects on all the family members (Knowles et al. 2010; Granroth-Wilding et al. 2015). Biparental care has been considered both, a cooperative behavior and a source of sexual conflict, as optimal levels of resource allocation are likely to differ between parents (Trivers 1972; Royle et al. 2004; Van Dijk et al. 2008; Mariette and Griffith 2015; Griffith 2019). Theoretical models on how parents should contribute to parental care typically predict that conflicts can be solved by partial compensation response strategies. Under partial compensation, biparental care is evolutionary stable because any decreased effort by one parent leads to reductions in fitness as partners only partially compensate (Houston et al. 1985; Webb et al. 1999; McNamara et al. 2003). Furthermore, theoretical elaborations predict that “negotiation” over parental care could lead to partial, full, or even no compensation (or desertion) depending on the shapes of the cost and benefit functions of parental care, the information that parents have on offspring needs (Johnstone and Hinde 2006), and among-individual differences in breeding strategies or condition (Harrison et al. 2009). For instance, in species with long-term pair bonds, or long-lasting parental care, the costs of decreased parental investment by one parent may be equally adverse for both parents, and patterns of investment that optimize the fitness of the pair over the long term may be expected (Griffith 2019). Hence, because mounting an immune response can influence the host physiology and parental contribution, the impact of infection could include indirect effects on the unchallenged mate’s contribution to parental care (Knowles et al. 2010; Granroth-Wilding et al. 2015). However, the potential consequences of mounting an immune response on the distribution of parental contribution to the offspring care have rarely been investigated (Granroth-Wilding et al. 2015).

The brown booby (*Sula leucogaster*) is a long-lived seabird with reversed sexual size dimorphism (females are roughly 33% heavier than males) and prolonged biparental care. Brown boobies are socially monogamous, with a low level of extra-pair copulations (Gañan et al. 2014). Mate fidelity between breeding events varies among colonies, and in the colony of the present study is low (Guillen-Parra et al. 2023). Both parents incubate on average for 42 d a modal clutch of 2 eggs, which hatch asynchronously with a 4-d interval. This hatching asynchrony creates a within-brood hierarchy that facilitates brood reduction typically during the first week of life, resulting in only one chick produced per breeding attempt (Osorno and Drummond 2003). Males and females feed and attend the offspring for roughly 3 mo (Nelson 1978; Tershy and Croll 2000). Feeding occurs in response to offspring begging behavior, which stimulates the direct mouth-to-mouth transfer of food (Nelson 1978). Interestingly, oxidative stress may be a cost and a constraint of reproduction as displaying a more intense gular color during courtship, a trait used in mate choice, is linked to greater levels of reactive oxygen species, and mothers with greater oxidative stress laid smaller first eggs and produced chicks with lower rates of mass gain (Montoya et al. 2016). Because of the prolonged and presumably costly biparental care, mounting an immune response during this demanding stage may impose physiological costs and compromise parental care decisions of the pair.

In the brown booby, we tested the hypothesis that activation of the immune response raises oxidative stress as an immediate cost and impacts how parental effort is shared by the immune-challenged parent and its mate. We conducted a field experiment in which one member of the pair (either the male or the female) was immune challenged during incubation by applying an intraperitoneal injection of lipopolysaccharide (hereafter LPS-treated parent), a strongly immunogenic molecule of *Escherichia coli*. We predicted that compared to parents in the control group, LPS-treated parents will show (1) an increase in oxidative stress parameters (oxidative damage to lipids and total antioxidant capacity). If mounting an immune response compromises parental behavior, we expected that (2) LPS-treated parents will spend less time attending the offspring, preen their offspring at lower rates, and have a lower feeding rate in response to the offspring begging than control-treated parents, and (3) that mates of LPS-treated parents will increase parental effort as a compensatory mechanism to avoid detrimental effects on offspring growth. Hence, under the scenario that unchallenged mates of LPS-treated parents compensate, we predicted (4) no significant differences in growth rate, oxidative stress, and immune response between offspring from LPS-treated and control parents.

## Methods

The study was conducted from July to September 2016 in the brown booby breeding colony at Isla Larga, Parque Nacional Islas Marietas, Nayarit, México (20° 41ʹ N 105° 36ʹ W).

### Experimental immune challenge

During incubation, we captured 61 pairs by night lighting, marked them individually with a numbered white polymethylmethacrylate leg band (Interrex, Poland), and their nest with a numbered flag. We randomly assigned pairs to the LPS group (*n* = 41) or the control group (*n* = 20). In the LPS group, one member of each pair (i.e. LPS-treated parent, females in 20 pairs and males in 21 pairs) was intraperitoneally injected with 0.10 mg of *E. coli* LPS (serotype O55:B5, Sigma Aldrich, catalogue L2637, St. Louis, MO, USA) dissolved in 1 mL of Phosphate Buffered Saline solution (PBS; Sigma Aldrich, catalogue P4417, St. Louis, MO, USA). In the control group, one member of each pair was sham-treated (i.e. control-treated parent, females in ten pairs and males in ten pairs) with an intraperitoneal injection of 1 ml of PBS. Ten days later, we applied a second LPS injection to the LPS-treated parents to activate a secondary humoral immune response and a second PBS injection to the control-treated parents. LPS injections were administered on average 28 ± 12 and 18 ± 12 d before hatching for the first and the second dose, respectively. LPS is a conserved pathogen-associated molecular pattern with a highly immunogenic component of Gram-negative bacteria cell walls that mimics a bacterial infection and has been widely used in ecoimmunological studies (Costantini 2022). Within hours to a few days, LPS exposure causes an acute phase response that includes an inflammatory response, sickness behaviors such as reduced locomotion and feeding, and an increase of oxidative stress markers (Torres and Velando 2007; Armour et al. 2020; Costantini 2022).

Although less studied, the effects of modulating the acute phase response after LPS-immune challenge can last longer (20 d in the Eurasian blackbirds, *Turdus merula*; Lennon et al. 2023), affecting key life history traits (e.g. migration or breeding investment; Kelly et al. 2020; Schoepf et al. 2022). Based on the body mass at first capture, the concentration of LPS injection was (mean ± SD) 0.10 ± 0.01 mg/kg of body mass (range 0.06 to 0.12 mg/kg). This is a relatively low concentration compared to those used in other bird experiments (0.01 to 5 mg/kg of body mass; Torres and Velando 2007; King and Swanson 2013; Butler et al. 2021; Montoya et al. 2023).

To determine oxidative stress parameters, we collected three 1.5 mL blood samples from the brachial vein of LPS- and control-treated parents: (1) before the first immune challenge (during the first capture), (2) 10 d (± 2 d) after the first challenge, before the second immune challenge (second capture), and (3) 7 d (± 2 d) after the second immune challenge (third capture). Immediately after collection, blood samples were kept on ice, plasma and blood cells were separated by centrifuging at 10 000 g × 10 min, and plasma was stored at −80 °C until laboratory analyses were performed. During each capture, we measured body mass (± 20 g) of adults. To determine the hatching date, the nests were checked daily between 14:00 and 16:00 h. This time period was chosen as it is the time of less activity, to avoid disturbing the colony. Bird handling took less than 12 min to minimize stress.

### Offspring growth and immune response

In the offspring, we measured the increase of body mass (± 10 g), tarsus, ulna, and beak length (± 0.1 mm) every 5 d from hatching until 30 d post-hatching. For oxidative stress analyses, we collected one 1 mL blood sample from 15-d-old offspring. Offspring blood samples were processed and stored as described above. As an estimate of the immune response of chicks, we used the phytohemagglutinin skin test that includes both, adaptive and innate components of the immune system (Martin et al. 2006). At the age of 30 d, offspring were injected subcutaneously in the left wing-web with 0.1 mL of 1 mg mL^−1^ PHA-P (Sigma Aldrich, catalogue L9016, St. Louis, MO, USA) dissolved in PBS. The point of injection was marked with an indelible marker and wing-web thickness was measured (3 times to yield an average) before injection and 24 h later with a Mitutoyo micrometer (± 0.001 mm). We estimated the immune response to PHA as the wing-web swelling 24 h after the injection. AMF performed all injections and measurements. The repeatability of swelling measures was high (intraclass correlation coefficient ± SE: before injection, *r = *0.96 ± 0.008, *P *< 0.001; after injection *r *= 0.99 ± 0.001, *P < *0.001).

### Behavioral observations

Observations of parental care started when the chicks were roughly 4 d old and lasted until the chicks reached an age of 30 d. Nests were observed every 5 d (± 3 d), from 7:00 to 9:30 h and 18:30 to 21:00 h, the periods of greater diurnal parental activity at the colony (Montoya and Torres 2015). The age of the chicks when behavioral observations started did not differ between the control (mean ± SE: 4.27 ± 0.23) and the LPS groups (4.52 ± 0.18, *F1,28 *= 0.74, *P *= 0.39). Behavioral records were conducted by 7 observers from roughly 3 to 6 m of distance from the focal nests. Observers were blind about which experimental treatment focal nests belonged. All nests recorded had only one chick after the second observation. Parental behaviors recorded were the following: (1) time that each parent spends near the chick (hereafter offspring attendance; recorded as the minute and hour a parent arrived and left the nest), (2) absolute number of feedings by each parent, (3) number of bouts of preening behavior from each parent to chick. From the focal offspring, we recorded (4) the number of begging bouts (when the chick raises the head and vocalizes with a “tac-tac” sound; a new begging bout was considered after a period of > 10 seconds of no begging; detailed descriptions of all recorded behaviors in Nelson 1978). We pooled all observations from each nest and calculated the time each parent spent with the offspring and the rate per hour of each parental behavior (preening rate = absolute number of preenings by each parent/total time the nest was observed; feeding rate = absolute number of feedings by each parent/total offspring attendance by the male or the female). We independently calculated begging rates for the mother and father (number of begging bouts to the male or the female/total offspring attendance by the male or the female). We started behavioral recordings after a period of observers training, when inter-observer reliability was high (*r *= 0.97 ± 0.02, *P *= 0.02).

### Oxidative stress measures

#### Protein quantification.

We used protein quantification as an estimation of the substrate available for redox reactions, as proteins per volume of plasma can vary among samples (Barja de Quiroga et al. 1991). Before analyses, plasma samples were diluted to a concentration of 0.1 in distilled water. In a 96-well microplate, we added 20 µL of Bradford solution (1 mL of Bradford reagent per 4 mL of distilled water) to 20 µL of standards and samples (by duplicate). The microplate was incubated at room temperature, without exposure to light for 3 min and was agitated for 30 s. The absorbance of the reaction was read at 620 nm using a microplate photometer (Thermo Scientific, Multiskan FC Microplate Photometer). The results were interpolated to a standard curve of serum bovine albumin (Sigma-Aldrich, catalogue 05470-1G, St. Louis, MO, USA). The average coefficient of variation (CV) between replicates was 5.71% within plates and 1.52% among plates.

#### Oxidative damage to lipids.

We estimated oxidative damage to lipids using the thiobarbituric acid reactive substances (TBARS) assay through the formation of the pink chromogen [TBA]2– malondialdehyde (MDA) adduct (Halliwell and Chirico 1993). MDA is one of the final products of polyunsaturated fatty acids peroxidation and the main advanced lipid oxidation product able to react with thiobarbituric acid (TBA; Halliwell and Chirico 1993). Briefly, we added 100 µL of trichloroacetic acid (10% v/v) to 100 µl of 0.1 diluted plasma and centrifuged to 3000 g × 10 min. If the supernatant was shady, we centrifuged it again at 3000 g × 5 min. About 180 µL of supernatant were recovered and 100 µL of thiobarbituric acid reagent were added (0.375% TBA and 2% acetic acid). The mixture was incubated at 92 °C × 45 min and then, it was placed on ice × 10 min to stop the reaction. After incubation, the absorbance of the pink chromogen TBA- MDA was measured at 530 nm using a microplate photometer (Thermo Scientific, Multiskan FC Microplate Photometer). A standard curve of MDA (1, 1, 3, 3-tetramethoxypropane, Sigma- Aldrich, catalogue 108383, St. Louis, MO) was used to estimate oxidative damage to lipids. Oxidative damage to lipids is expressed as nmol MDA mg^-1^ protein. All the samples from family members were analyzed in the same plate by duplicate. The average CV between replicates was 6.90% within plates and 6.14% among plates.

#### Total antioxidant capacity.

 We estimated plasma total antioxidant capacity by a quantitative colorimetric assay (QuantiChrom Kit, BioAssay Systems, Hayward, CA). By this method, Cu^2+^ is reduced by antioxidants present in the sample to Cu^+^. The resulting Cu^+^ forms a colored complex with a dye reagent, and color intensity is proportional to the total antioxidant capacity of the sample. In a 96-well microplate, we added 50 µL of working solution to 10 µl of curve standards and 0.1 diluted samples. The plate was incubated at room temperature, without exposure to light × 10 min, and the absorbance at 570 nm was read with a microplate photometer (Thermo Scientific, Multiskan FC Microplate Photometer). A standard curve of Trolox was used to calculate the total antioxidant capacity in the samples. Plasma total antioxidant capacity is expressed as mmol Trolox equivalents mg^−1^ protein. All the samples from family members were analyzed in the same plate by duplicate. The average CV between replicates was 7.78% within plates and 6.79% among plates.

### Statistical analysis

Sample sizes for different analyses may vary due to natural causes of breeding failure (nest desertion, predation) and missing samples during fieldwork (we were unable to re-capture all the focal birds in the study), and laboratory analyses (available plasma volume was insufficient to determine both oxidative stress parameters in some samples). From the original 61 nests, during incubation 36% (15/41; 6 males and 9 females) LPS- and 20% (4/20; 4 males) control nests were lost. The probability of nest failure by LPS and control pairs did not differ (logistic regression with binomial distribution of errors, χ^2^ = 1.67, *P *= 0.19, *n* = 61). In Marietas Islands, the proportion of nests that fail during incubation shows a strong interannual variation (11% (6/54) in 2011, 51% (25/49) in 2018, 46% (26/56) in 2019; data from unmanipulated nests, R. Torres, unpublished data). Hence, in this study, the overall proportion of failed nests (31%) was within natural variation. In addition, eggs did not hatch in 6 LPS (4 LPS-treated males and 2 LPS-treated females) and 4 control (2 control-treated males and 2 control-treated females) nests. Finally, one offspring from the LPS group and one from the control group died during the first week after hatching. Hence, initial sample for analyses of the effect of LPS immune challenge on the oxidative status of the parents was 61 nests (41 LPS and 20 control). The sample size for behavioral analyses was 30, 19 LPS (9 LPS-treated males and 10 LPS-treated females) and 11 control (4 control-treated males and 7 control-treated females) nests. Before the immune challenge, nests from the control and LPS treated groups did not differ in clutch size (χ^2^ = 1.83, *P *= 0.18), date of first capture (*F*1,59 = 2.74, *P *= 0.10), parental body mass (treatment *F*1,57 = 0.21, *P *= 0.65, sex *F*1,57 = 79.72, *P *< 0.001, treatment × sex *F*1,57 = 0.02, *P *= 0.88), oxidative damage to lipids (treatment *F*1,50 = 0.08, *P *= 0.78, sex *F*1,50 = 1.46, *P *= 0.23, treatment × sex *F*1,50 = 0.04, *P *= 0.84), or total antioxidant capacity (treatment *F*1,50 = 0.03, *P *= 0.87, sex *F*1,50 = 1.84, *P *= 0.18, treatment × sex *F*1,50 = 0.36, *P *= 0.55).

For the analyses to evaluate the effect of the treatment on oxidative stress parameters (oxidative damage to lipids and total antioxidant capacity), we used mixed-effects models. Oxidative damage to lipids and total antioxidant capacity were ln transformed before statistical analyses. Models to evaluate the effects of treatment on oxidative stress parameters of treated parents included as fixed effects: the treatment (LPS or Control), capture order (capture 1, 2, 3), and the sex of parent; and the nest identity as a random factor to control for the non-independency of repeated measures belonging to the same parent. Because oxidative damage to lipids is expected to be related to levels of total antioxidant capacity (Skrip and Mcwilliams 2016), this variable was included as a covariate in the analysis of oxidative damage to lipids.

To evaluate the effect of the treatment on parental contribution to offspring feeding, we used 3 general linear models with normal error distribution. The first model evaluated the effect of the treatment on the feeding rate of treated parents and included the treatment (LPS or control), the offspring begging rate, and its interaction with treatment; offspring begging elicits parental feeding and serves as a proxy of the chick’s level of hunger. Because males and females did not differ in feeding rate (mixed-effects model, sex *F*1,55 = 0.60, *P *= 0.44, begging *F*1,55 = 22.88, *P *< 0.001, sex × begging *F*1,55 = 1.53, *P *= 0.22), to reduce the number of parameters included in the model, the sex of the parent was dropped from the analysis of feeding rate. Second, to evaluate if mates of LPS-treated parents increase their feeding rate as a compensatory mechanism when the feeding rate of treated parents declines, we ran a model that included as response variable the difference within-pair in the rates of feeding (feeding rate of the mate—feeding rate of treated parent), and as explanatory variables the treatment (LPS or control), the difference within-pair of the offspring begging rate to the treated parent or the mate (begging rate to mate—begging rate to treated parent) and the interaction between these 2 terms. Third, to evaluate the overall feeding rate by pairs in the LPS and control groups, we used a linear model that included as response variable the overall feeding rate by both members of the pair as the response variable, and as explanatory variables the treatment (LPS or control), the overall begging rate by chicks and the interaction between treatment and begging rate.

To evaluate the effect of the treatment on the rate of preening and the time that the parents attend the offspring, we used independent mixed-effects models that included the nest identity as a random factor to control for the non-independency of parents belonging to the same nest, and treatment (LPS or control), parent (treated-parent or untreated mate), sex of parent, and the interaction treatment × parent as fixed effects. To evaluate if the overall preening (sum of preening rate by both members of the pair) received by the offspring in the LPS and control groups differed we used a linear model. Variation among individuals in the number of days between LPS application and hatching date was unrelated to feeding rate (LM test: *F*1,27 = 0.45, *P *= 0.50), preening rate (LM test: *F*1,28 = 0.14, *P *= 0.71), and the time the parents spent on the nest (LM test: *F*1,28 = 0.36, *P *= 0.55). Thus, the variable days from LPS application to hatching date were not included in the analyses.

Offspring daily increase in mass, beak, and ulna was calculated as the difference between the final (age 30 d) and first (age 1 d) measurements, divided by the number of days that chick growth was recorded. To summarize the growth of chicks, we used a Principal Component Analysis (based on a correlation matrix) that included the daily rates of increase of mass, beak, and ulna that resulted in only one Principal Component (eigenvalue = 2.01, variance explained 67%). PC1 (hereafter, offspring growth) loading coefficients were mass increase of 0.878, beak increase of 0.744, and ulna increase of 0.829. We used linear models to analyze the effect of the treatment to parents on the offspring growth, oxidative damage to lipids, total antioxidant capacity, and wing-web swelling in response to the PHA test.

Analyses were carried out with R 4.2.2 (R Core Team 2022) using RStudio 2022.12.0 + 353 (RStudio Team 2023) package “nlme” (Pinheiro et al. 2017). For mixed-effects models we estimated marginal *R*^2^ (proportion of total variance explained by fixed effects) and conditional *R*^2^ (proportion of total variance explained by the fixed and random effects) values for each model (Nakagawa and Schielzeth 2013). Final models were obtained by stepwise backward deletion of nonsignificant terms (*P *> 0.05). Outliers were tested by the Bonferroni test using the outlierTest function from package “car” (Fox et al. 2012). All figures were created using the package “ggplot2” (Wickham 2016).

## Results

### Does an immune challenge increase oxidative stress?

**Fig. 1.**
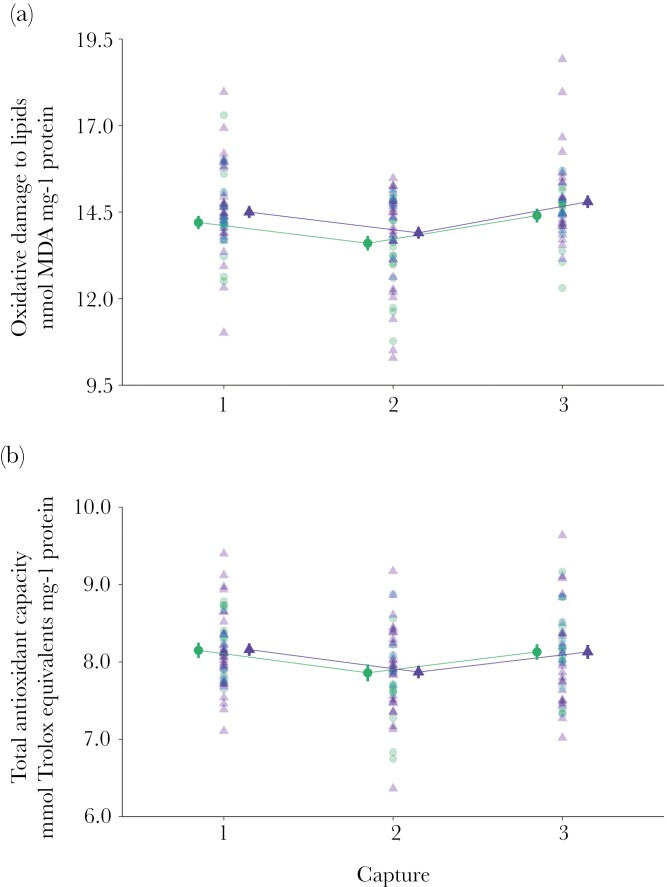
Mounting an immune response during incubation did not affect levels of (a) oxidative damage to lipids or (b) total antioxidant capacity. To activate the immune system individuals were immune challenged with 2 intraperitoneal injections of lipopolysaccharide (LPS-treated parents), while individuals in the control group received 2 injections of PBS (control-treated parents). Injections were administrated during the first and the second capture, mean intervals between captures were 10 and 7 d, respectively. Figures show estimated mean ± SE and individual scores for LPS-treated parents (triangles) and for control-treated parents (circles).

Overall, oxidative damage to lipids of treated parents was not affected by the treatment but varied over time (estimated marginal means ± SE; capture 1: 14.40 ± 0.15, capture 2: 13.80 ± 0.16, capture 3: 14.60 ± 0.17; Table 1; Fig. 1a). Also, the levels of oxidative damage to lipids varied along with the levels of total antioxidant capacity (β = 0.95 ± 0.12) and did not differ between males and females (Table 1). Similarly, levels of total antioxidant capacity of treated parents varied over time (estimated marginal means ± SE; capture 1: 8.23 ± 0.09, capture 2: 7.94 ± 0.09, capture 3: 8.18 ± 0.09), but neither the treatment nor the sex of the parent explained this variation (Table 1; Fig. 1b).

**Table 1.** Effects of LPS treatment on oxidative damage to lipids and total antioxidant capacity of treated parents. Linear mixed-effects models fit included nest identity as a random factor, and treatment (control or LPS), sex of the parent, capture (first capture [first LPS challenge], second capture 10 d later [second LPS challenge], and third capture 7 d later) and second and third order interactions as fixed effects. Non-significant interactions were eliminated from models.

*Predictors*	*Oxidative damage to lipids*			Total antioxidant capacity		
	*df*	*F*	*P*	*df*	*F*	*P*
Treatment	**1,49**	**3.01**	**0.09**	**1,52**	**0.005**	**0.94**
Sex	**1,49**	**2.57**	**0.11**	**1,52**	**0.23**	**0.63**
Capture	**2,93**	**7.22**	**0.001**	**2,92**	**5.53**	**0.005**
Total antioxidant capacity	**1,128**	**50.25**	**<0.001**			
***Excluded terms***						
Capture × Treatment	2,134	1.42	0.24	2,77	0.13	0.88
Capture × Sex	1,132	0.36	0.70	2,79	2.03	0.14
Treatment × sex	1,131	0.00	0.99	1,37	0.03	0.87
Capture × treatment × sex	2,129	0.06	0.94	2,76	0.64	0.53
N (nests/observations)	56/137			56/144		
Marginal *R*^2^/Conditional *R*^2^	0.38/0.41			0.04/0.33		

Effect size is reported as marginal *R*^2^ (variance explained by fixed effects) and conditional *R*^2^ (variance explained by fixed and random effects). Variables in the final model are shown in bold.

### Does an immune challenge compromise parental effort?

**Fig. 2.**
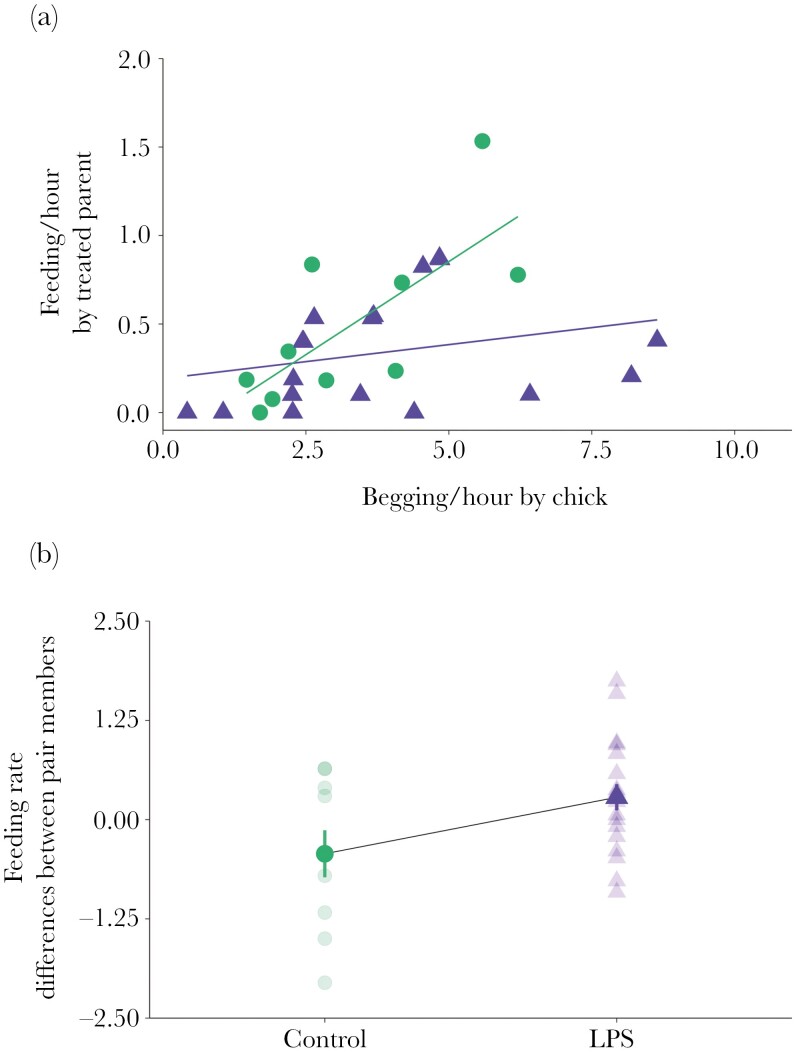
Mounting an immune response decreased feeding rate of LPS-treated parents and increased feeding rate of the LPS mates. (a) Feeding rate of LPS- and control-treated parents as a function of chick begging rate. (b) Within-pair differences in feeding rate of LPS and control pairs; figure shows estimated mean ± SE and individual scores for LPS-treated parents and their mates (triangles) and control-treated parents and their mates (circles).

During the first month post-hatching, LPS-treated parents responded to offspring begging with a feeding rate 45% lower compared to control-treated parents (estimated marginal means ± SE; LPS 0.55 ± 0.11, control 1.00 ± 0.17; Table 2A; Fig. 2a). In addition, on average, LPS mates had a greater feeding rate than their LPS-treated partners, compared to the differences in feeding rate between control mates and their control partners (Table 2B; Fig. 2b). Also, there was a positive relationship between the differences in feeding rate between pair members and the differences in offspring begging to the treated parent or the mate (β* *= 0.43, *t *= 2.59, *P *= 0.01; Table 2B). Hence, despite the decline in feeding rate by LPS-treated parents, overall feeding rate by the pair in LPS and control nests did not differ (estimated marginal means ± SE: LPS-treated nest 0.61 ± 0.09, control-treated nest 0.68 ± 0.13; Table 2C).

**Table 2.** Effects of LPS exposure on parental contribution to offspring feeding. (A) Analysis of the feeding rate (h) of LPS-treated and control-treated parents. (B) Analysis of within-pair differences in feeding rate of LPS and control pairs. (C) Analysis of total pair feeding rate (h). Linear models were used for the analyses and variables in the final models are shown in bold.

Variables	*df*	*F*	*P*
A. Feeding rate of treated parents			
**Treatment**	**1,25**	**4.83**	**0.04**
**Begging rate**	**1,25**	**11.77**	**0.002**
**Treatment × begging rate**	**1,25**	**8.47**	**0.007**
N = 29, Adjusted R^2^ = 0.26			
B. Pair differences in feeding rate			
**Treatment**	**1,25**	**4.15**	**0.05**
**Difference in begging rate between parents**	**1,25**	**6.73**	**0.02**
Treatment × difference in begging rate	1,24	3.02	0.09
N = 29, Adjusted R^2^ = 0.33			
C. Total pair feeding			
**Treatment**	**1,26**	**0.17**	**0.68**
**Begging rate**	**1,26**	**17.65**	**< 0.001**
Treatment × begging rate	1,25	0.83	0.37
N = 29, Adjusted R^2^ = 0.37			

**Fig. 3.**
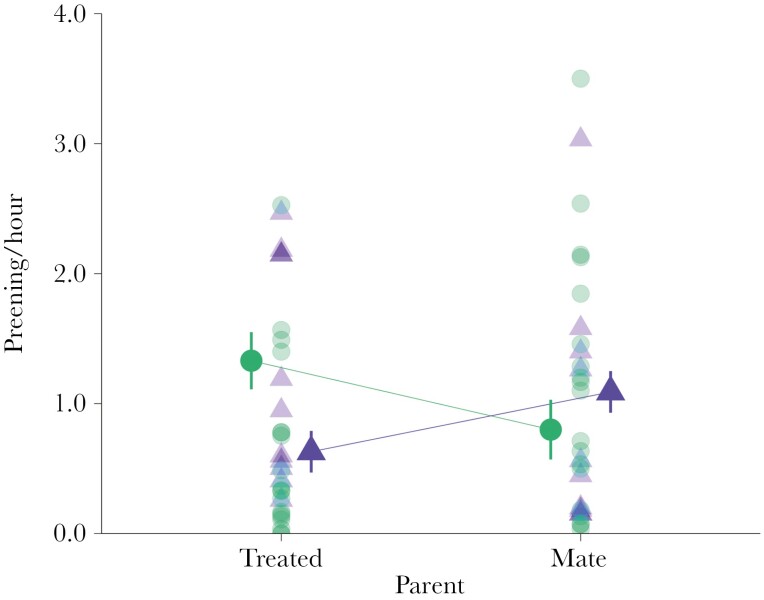
LPS-treated parents had a lower rate of preening than control-treated parents. Figure shows estimated mean ± SE and individual scores for LPS-treated parents (triangles) and control-treated parents (circles).

The immune challenge affected the rate at which parents preen their offspring (Table 3, Fig. 3). LPS-treated parents preen the offspring at a rate 53% times lower than control parents (estimated marginal means ± SE: LPS-treated parent 0.63 ± 0.16 vs control-treated parent 1.33 ± 0.22, post hoc test: *t* ratio = −2.51, *P *= 0.01), and 42% lower than LPS mates, but this difference did not reach significance (LPS mates 1.09 ± 0.16, *t* ratio = −1.91 *P *= 0.06; Fig. 3). The rate of preening by control-treated parents and their mates did not differ (control-treated parent 1.33 ± 0.22, control mate 0.80 ± 0.23 post hoc test: *t* ratio = 1.63 *P *= 0.11; Table 3, Fig. 3).

**Table 3.** Effects of LPS treatment on offspring preening and nest attendance by treated parents and their untreated mates. Linear mixed-effects models fit included nest identity as a random factor and treatment (control or LPS), parent (treated-parent or untreated mate) and the sex of the parent as fixed effects.

*Predictors*	*Offspring preening*			Nest attendance		
	*df*	*F*	*P*	*df*	*F*	*P*
Treatment	**1,54**	**1.08**	**0.30**	**1,28**	**0.06**	**0.80**
Parent (treated or mate)	**1,54**	**0.03**	**0.86**	**1,28**	**1.11**	**0.30**
Sex	**1,54**	**20.64**	**<0.001**	**1,28**	**4.32**	**0.05**
Treatment × parent	**1,54**	**6.05**	**0.02**	1,27	1.60	0.22
N (nests/observations)	30/59			30/60		
Marginal *R*^2^/Conditional *R*^2^	0.31/0.31			0.06/0.31		

Effect size is reported as marginal *R*^2^ (variance explained by fixed effects) and conditional *R*^2^ (variance explained by fixed and random effects). Variables in the final model are shown in bold.

In addition, males preen their offspring at a higher rate than females (estimated marginal means ± SE: males 1.41 ± 0.14, females 0.53 ± 0.14, post hoc test: *t* ratio = −4,54, *P = *0.001). Overall, the total preening rate by pairs from the LPS and control group did not differ (estimated marginal means ± SE: LPS nests 1.73 ± 0.22, control nests 2.14 ± 0.30, treatment *F*1,27 = 1.20, *P *= 0.28).

Exposure to LPS was unrelated to the time parents spent at the nest (Table 3). Overall, males tend to spend on average 9% more time at the nest than females (Table 3).

### Does an immune challenge on parents impair offspring growth, oxidative status, or immune response?

Having a parent that was immune challenged did not affect offspring growth during the period of rapid growth (treatment, *F*1,28 = 0.25, *P *= 0.62), the levels of oxidative damage to lipids (treatment, *F*1,24 = 1.40, *P *= 0.24), or the levels of total antioxidant capacity (treatment, *F*1,26 = 1.69, *P *= 0.20) at the age of 15 d, neither the offspring immune response to PHA (treatment, *F*1,25 = 1.75, *P *= 0.19).

## Discussion

Mounting an immune response is expected to impose physiological costs such as increased oxidative stress and may compromise parental effort. In the brown booby, we found no evidence that mounting an immune response increases oxidative damage to lipids or total antioxidant capacity. However, parents who were immune challenged during incubation showed a decline in their contribution to parental duties. Interestingly, the decline in parental effort of LPS-treated parents did not result in poorer offspring growth, possibly due to parental care compensation by mates.

Contrary to our prediction, we did not detect an increase in oxidative stress markers in LPS-treated parents compared to control-treated parents. Oxidative markers are commonly reported to increase in response to an immune challenge, but this response is not ubiquitous in wild animals (Cram et al. 2015; Costantini 2022). Oxidative stress is a dynamic complex process, and the effects of mounting an immune response may have impacted other markers not measured here or within a timeframe out of the scope of our sampling scheme. Alternatively, it is possible that between our sampling points, LPS-treated parents could have minimized the oxidative effects of the immune insult by up-regulating antioxidant defenses, repair mechanisms, or by modulating the immune response (Costantini 2014; Morris et al. 2022), or through compensatory behavior, decreasing investment in costly activities (Bonneaud et al. 2003; Adelman and Martin 2009). In a sister species, the blue-footed booby, an LPS immune challenge during courtship resulted in an age-dependent effect on oxidative markers and reproductive effort: compared to controls, mature males (< 10 yr) that were immune challenged showed no significant variation in oxidative markers and a decline in the number of fledglings produced, whereas senescent males (> 10 yr) had increased levels of oxidative damage to lipids, and a 98% increase in reproductive output (Velando et al. 2006; Torres and Velando 2007). In the present study, age of the experimental birds was unknown, but if mature individuals were overrepresented in our sample, the apparent lack of oxidative costs in immune-challenged birds may result from a strategy of favoring investment in self-maintenance over reproduction, as LPS-treated parents also declined their parental effort. In our study, oxidative damage to lipids and total antioxidant capacity from LPS- and control-treated birds covaried through time, declining after the first injection and then, increasing after the second one. Hence, regardless of the treatment received (LPS or control), oxidative damage to lipids and total antioxidant capacity declined through the incubation period, and then, increased as hatching approached, suggesting that physiological and behavioral changes associated with the breeding stage impact the oxidative status of brown boobies as has been previously suggested (Montoya et al. 2016).

In the brown booby, fighting an infection during the incubation period impaired parental effort during the chick-rearing period. We found that the activation of the immune system resulted in roughly a 50% decline in the rate of feeding in response to begging and in the rate of preening of LPS-treated parents compared to control-treated parents. The immune challenge was unrelated to nest attendance by parents. In free-living birds, reduced activity after an immune challenge with LPS has been reported to last up to 20 d (Lennon et al. 2023). Interestingly, the extent and duration of these lasting effects may vary with social and environmental conditions, as well as with the individual physiological condition and its life history stage. In our study, birds were immune challenged with a relatively low dose of LPS, however, changes in parental behavior were detectable on average 48 d after the challenge, indicating that the effect of mounting an immune response during reproduction, an energetically demanding life history stage, can have longer effects than previously reported. During parental care, investment in feeding effort can be costly to parents in terms of energy and nutrients and may compromise physiological processes, future survival, and reproduction (Sheldon and Verhulst 1996; Lochmiller and Deerenberg 2000; Monaghan et al. 2009). Also, preening, a time-consuming behavior, has an important role in the control of ectoparasites, which may influence offspring growth and survival (Fitze et al. 2004; Bush and Clayton 2018; Romano et al. 2021). Therefore, individuals might not be able to maximize simultaneously immune defense and reproductive effort (Sheldon and Verhulst 1996). Furthermore, during immune defense, a decline in parental effort might be adaptive if it allows the individual to overcome infection and increase survival prospects. Also, a reduction in the feeding behavior may decrease the risk of disease transmission from parents to offspring (Shakhar and Shakhar 2015). Our results provide further evidence for the proposal that one of the costs of immune response to infections is a decline in parental effort (Ilmonen et al. 2000; Råberg et al. 2000; Bouslama et al. 2002; Gallizzi et al. 2008).

Exposure to LPS by one parent affected the feeding rate and possibly the preening rate of the untreated mate. LPS mates had a relatively greater feeding rate than their partners compared to pairs in the control group. Also, the preening rate of mates of LPS-treated parents had a greater preening rate than their partner but the difference did not reach significance. The results are consistent with the idea of parental care compensation in response to a decline in the mate’s parental effort. Long-lived species tend to remain more genetically faithful to one another favoring behaviors of mate compensation or cooperation (Griffith 2019). In the brown booby extra-pair copulations are relatively low (Gañan et al. 2014) and because of the long-lasting parental care, deserting the nest at the stage of chick rearing is possibly a costly strategy, as remating with a new partner in the same breeding season is unlikely. We do not know how brown boobies evaluate the feeding (or preening) effort of their mates to adjust their own effort. One possible mechanism for feeding effort compensation is that after the decline in food provisioning by one parent, offspring increased begging behavior eliciting a greater feeding rate from the mate. Accordingly, we found that mates’ feeding rate was greater compared to the treated parent when offspring increased begging toward the mate (i.e. a positive relationship between the within-pair difference in feeding and offspring begging rate to the treated parent and the mate). Future studies should investigate in more detail the interaction between chick begging, parental provisioning, and negotiation between mates over parental feeding, and whether parents may glean information about the need for parental care from the behavior and/or condition-dependent signals of their mates to adjust their own provisioning (Johnstone and Hinde 2006). Independent of the mechanism used by the brown booby to evaluate the mates’ parental effort, our results suggest that as expected in long-lived species that tend to remain more genetically faithful, mate compensation may be favored (Griffith 2019).

As expected under the scenario of parental compensation the decline in parental effort by LPS-treated parents did not impair offspring growth or physiological condition. Offspring from control and LPS-treated parents did not differ in daily growth rate, oxidative stress markers (oxidative damage to lipids and total antioxidant capacity), and immune response to PHA. The lack of negative effects of the LPS treatment on the offspring supports the interpretation that mates compensated for reduced partner effort (Harrison et al. 2009; Griffith 2019).

Mounting an immune response is expected to be particularly costly during energetically demanding life-history stages such as reproduction and may result in shifts in resource allocation (e.g. Knowles et al. 2010; Schoepf et al. 2022). When fighting an infection, animals in addition to the activation of the immune response, modify their behavior by reducing their activity to diverge resources toward immune defense. Intensity and duration of the sickness behavior are expected to vary according to the intensity of the infection, the host condition, and social context, potentially leading to changes in the breeding strategy. Hence, in species with biparental care, the indirect costs of fighting an infection may influence how the pair negotiates the division of parental duties, with potential effects on present and future reproduction. Future studies should consider that the effects of fighting an infection could extend beyond an individual host (Lopes 2023).

In conclusion, in the brown booby, an immune challenge during incubation did not increase oxidative stress parameters, yet challenged individuals diminished their parental care, and their mates compensated for this decline, resulting in no identified detrimental effects in the offspring. These results are consistent with the idea that LPS-treated parents switched their strategy to favoring self-maintenance over reproduction. Furthermore, our results suggest that particularly in species with biparental care where both pair members benefit from the output of the breeding event, family relationships are likely to play a role in the response and fitness consequences of host-parasite interactions (Granroth-Wilding et al. 2015). These results highlight the indirect effects of fighting an infection, eliciting behavioral adjustments in other family members to mitigate the negative effects of parasites.

## Data Availability

Analyses reported in this article can be reproduced using the data provided by Martinez-Flores et al. (2024).
